# Comment on “Differential synovial fluid white blood cell count for the diagnosis of chronic peri-prosthetic joint infection – a systematic review and meta-analysis” by Sabater-Martos et al. (2025)

**DOI:** 10.5194/jbji-10-335-2025

**Published:** 2025-09-05

**Authors:** Pim W. van Egmond, Olav P. van der Jagt, Jesse W. P. Kuiper

**Affiliations:** 1 Department of Orthopedic Surgery, ETZ Hospital, 5022 GC Tilburg, the Netherlands; 2 Department of Orthopedic Surgery, Martini Hospital, 9728 NT Groningen, the Netherlands

With great interest, we read the recent and commendable paper by Sabater-Martos et al. (2025). The authors conducted an impressive and comprehensive systematic review and meta-analysis evaluating the diagnostic accuracy of synovial white blood cell (WBC) counts and polymorphonuclear neutrophil (PMN) proportions for chronic periprosthetic joint infection (PJI). Importantly, they established optimized diagnostic thresholds and examined performance under complex clinical scenarios. They concluded that synovial fluid analysis remains a critical diagnostic tool for chronic PJI – a conclusion that we fully support.

Diagnosing chronic PJI remains notoriously challenging. The Musculoskeletal infection Society (MSIS), European Bone and Joint Infection Society (EBJIS), and International Consensus Meeting (ICM) 2018 criteria are currently the most widely used diagnostic reference standards, combining clinical, laboratory, microbiological, and histological findings. While robust, these criteria do not represent a universally accepted gold standard. The work by Sabater-Martos et al. (2025) is valuable in this context, synthesizing evidence across 72 studies to clarify diagnostic performance and optimal thresholds.

That said, we would like to highlight a persistent methodological issue in many diagnostic accuracy studies. One that, understandably, finds its way into meta-analyses like this. Because intraoperative findings are integral to PJI diagnosis, many studies are based solely on revision arthroplasty cohorts. While these patients undergo full evaluation, this introduces selection bias: not all painful arthroplasties are revised, and some unrevised cases may still be infected. Some studies attempt to address this by including patients who receive a full preoperative work-up but are not revised. However, in these cases, intraoperative data are unavailable, and potential false negatives may go unrecognized. This issue is present in at least three studies included in the meta-analysis (Berger et al., 2017; Lenski and Scherer, 2014; Wang et al., 2023).

To illustrate this issue, we ask the reader to consider 1500 painful arthroplasties evaluated with a standardized preoperative protocol, including joint aspiration. Of these, 500 are not revised and are assumed to be uninfected. However, some might be infected but detectable only intraoperatively. The remaining 1000 revised patients undergo full assessment. Calculating test accuracy across all 1500 patients may inflate sensitivity due to undetected false negatives. Conversely, analysing only the 1000 revised cases may yield more accurate performance but only for that selected group.

In a prior study on the alpha defensin lateral flow test for chronic hip PJI, we referred to non-revised painful hips as “Schrödinger hips”: a reference to the thought experiment illustrating uncertainty until observation (Kuiper et al., 2022). Similarly, a prosthetic joint's infection status remains unknowable without surgery.

A potential solution is differential verification: revised patients are evaluated with intraoperative criteria, while non-revised patients receive the same preoperative work-up and are followed over time. Wang et al. (2023) used this approach with a 1-year follow-up, reducing verification bias (Wang et al., 2023). Extending follow-up to at least 2 years – ideally 4–5 years – may yield even more reliable results.

While we are confident that the authors' conclusions remain valid, even considering these limitations, recognizing the potential for selection bias and underestimated false negatives remains crucial when interpreting test accuracy for chronic PJI and might help in designing future diagnostic studies for chronic PJI.
